# Google Trends and Injectable Products: The Next-Best Tool for Anticipating Patient Concerns in Plastic and Reconstructive Surgery

**DOI:** 10.1055/s-0043-1762914

**Published:** 2023-03-28

**Authors:** Mallory A. Rowley, Kometh Thawanyarat, Jennifer K. Shah, Rahim Nazerali

**Affiliations:** 1 State University of New York, Upstate Medical University, Syracuse, New York; 2 Medical College of Georgia at Augusta University, AU/UGA Medical Partnership, Athens, Georgia; 3 Stanford University, Vice Provost for Undergraduate Education, Stanford, California; 4 Division of Plastic and Reconstructive Surgery, Stanford University School of Medicine, Stanford, California

Professional health care organizations including the American Society of Plastic Surgeons (ASPS) historically provide guidance to medical providers in the wake of major medical news and events. Consider, for example, the ASPS guidance published in response to the December 17, 2020 Food and Drug Administration (FDA) briefing document of the Vaccines and Related Biological Products Advisory Committee for the Moderna COVID-19 (coronavirus disease 2019) Vaccine. 1 This information provides a framework for physicians to guide patient-physician conversations in formalized health care settings. However, it provides little insight into tangible patient concerns during the contemplation stage of decision-making prior to formalized patient consultations. We suggest utilizing Google Trends (GT) as a real-time tool to aid in identifying patient concerns.

There have been several previously published case reports commenting on the immunogenicity of hyaluronic fillers as well as COVID-19 vaccine hypersensitivity and dermal manifestations. 2 3 4 5 In theory, statements from professional organizations have a broader audience base than research articles from independent bodies including research institutions and universities. Few tools are available to both analyze how the public perception changes and capture significant concerns in the wake of these statements. In this virtual era where health-related information is readily accessible to patients via Internet searches, patients often form biases and premature decisions regarding treatment options prior to ever setting foot—or meeting virtually—in a provider's office. 6 7 Additionally, there is an ever-growing body of literature supporting the use of social media and its utility in communicating with patients, particularly within the field of plastic and reconstructive surgery. 8 9 10

In order for physicians to address patient concerns on their Web-based platforms in the absence of in-person patient consultations, we advocate for the use of free analytical tools, particularly GT, to predict and mitigate patient concerns. We used a GT analysis to examine whether public interest in and concern over dermal fillers and related injectable products, namely botulinum toxin, increased in the wake of FDA adverse event reports, specifically the aforementioned December 2020 FDA Briefing.

In our analysis, the following search terms ( Table 1 ) were queried on GT (Google LLC., Mountain View, CA). These search terms were grouped by the following categories: general terms, adverse reaction terms, safety consideration terms, procedural terminology, brand names of botulinum toxin, and brand names of dermal fillers. These search terms were chosen to capture the various aspects of patient concerns regarding these products, their uses, and safety with respect to the COVID-19 vaccine.

**Table 1:** Search terms related to cosmetic injectables, safety, and the COVID-19 vaccine used in the Google Trends query

Category	Search terms used
General terms	botox COVID botox vaccine dermal filler vaccine facial fillers COVID fillers COVID fillers vaccine lip fillers COVID lip filler vaccine
Adverse reaction terms	botox allergic reaction botox reaction fillers allergic reaction fillers reaction
Safety consideration terms	botox safety botox dangerous
Procedural terminology	botox dermal fillers facial fillers
Brand names for botulinum toxin	Botox Dysport Dysport botox Jeuveau Jeuveau botox Xeomin Xeomin botox
Brand names for dermal fillers	Aquamid Bellafill Captique Hylaform Juvéderm Juvéderm dermal filler Perlane Prevelle Puragen Radiesse Radiesse dermal filler Restylane Restylane dermal filler Sculptra Sculptra dermal filler

Abbreviation: COVID-19, coronavirus disease 2019.

The search terms were gathered from November 1, 2019 to November 1, 2021. These dates provided a year's worth of data before and after the inflection point—November 1, 2020—which was chosen to capture the media coverage leading up to the official FDA statement released in December 2020. GT normalizes the volume of the search terms to compare them against one another. Thus, the data extracted is assigned a value from 0 to 100 to represent the relative search volume for each term.

Once the data had been extracted from the GT platform for each search term, a bivariate regression analysis of panel data was utilized to explore whether or not the search term volumes were significantly increased after the chosen inflection point of November 1, 2020, as compared with the search volume prior to the inflection point. The data inputs were grouped by search volume for each term up to the inflection point of November 1, 2020, and search volumes for each term following the inflection point. Significant increases or decreases were statistically determined with a *p* -value of < 0.05 at a 95% confidence interval. Table 2 demonstrates that general terms ( *p* < 0.001), safety consideration terms ( *p* = 0.034), procedural terminology ( *p* < 0.001), brand names for botulinum toxin ( *p* < 0.001), and brand names for dermal fillers ( *p* = 0.049) had significantly different relative search volume before versus after the chosen inflection point of November 1, 2020.

**Table 2:** Bivariate regression results for cosmetic injectables, safety, and the COVID-19 vaccine search terms

Category	Coefficient	Standard error	*z* -score	*p* > | *z* |	95% CI
General terms	1.789	0.350	5.10	**< 0.001**	(1.102–2.476)
Adverse reaction terms	0.324	0.168	1.92	0.054	(–0.006 to 0.653)
Safety consideration terms	0.216	0.102	2.12	**0.034**	(0.016–0.417)
Procedural terminology	0.816	0.104	7.86	**< 0.001**	(0.612–1.019)
Brand names for botulinum toxin	1.583	0.265	5.97	**< 0.001**	(1.063–2.102)
Brand names for dermal fillers	0.486	0.247	1.97	**0.049**	(0.003–0.970)

Abbreviations: CI, confidence interval; COVID-19, coronavirus disease 2019.

Our GT analysis demonstrated a significant increase in search terms, and therefore public interest, related to injectable products, the COVID-19 vaccine, and their adverse effects following the December 2020 FDA Briefing. GT analysis allows providers to accurately query public interest in, and concern over, medical procedures in the absence of face-to-face, individual patient meetings. Using free, publicly available analytical tools allows physicians the ability to tailor patient education on social media or other Web-based platforms to anticipate and mitigate patient concerns.

## References

[1] Zhang R FDA briefing document of the Vaccines and Related Biological Products Advisory Committee for the Moderna COVID-19 VaccineFda.gov. Published 2021. Accessed December 29, 2021 at:https://www.fda.gov/media/144585/download

[2] Bhojani-Lynch T Late-onset inflammatory response to hyaluronic acid dermal fillers Plast Reconstr Surg Glob Open 2017 5 12 e1532 29632758 10.1097/GOX.0000000000001532 PMC5889432

[3] Gambichler T Boms S Susok L Cutaneous findings following COVID-19 vaccination: review of world literature and own experience J Eur Acad Dermatol Venereol 2022 36 02 172 180 34661927 10.1111/jdv.17744 PMC8656409

[4] McMahon D E Amerson E Rosenbach M Cutaneous reactions reported after Moderna and Pfizer COVID-19 vaccination: a registry-based study of 414 cases J Am Acad Dermatol 2021 85 01 46 55 33838206 10.1016/j.jaad.2021.03.092 PMC8024548

[5] Michon A Hyaluronic acid soft tissue filler delayed inflammatory reaction following COVID-19 vaccination - a case report J Cosmet Dermatol 2021 20 09 2684 2690 34174156 10.1111/jocd.14312 PMC8447415

[6] Birkeland S Moulton B Shared decision-making and liability in aesthetic surgery Aesthet Surg J 2016 36 08 NP254 NP255 27466600 10.1093/asj/sjw067

[7] Ubbink D T Santema T B Lapid O Shared decision-making in cosmetic medicine and aesthetic surgery Aesthet Surg J 2016 36 01 NP14 NP19 26104476 10.1093/asj/sjv107

[8] Cohen S A Tijerina J D Amarikwa L Men C Kossler A #PlasticsTwitter: The Use of Twitter Data as a Tool for Evaluating Public Interest in Cosmetic Surgery Procedures[published online ahead of print, 2021 Dec 28]Aesthet Surg J 2022 42 05 NP351 NP360 34962572 10.1093/asj/sjab429

[9] Rohrich R J Savetsky I L Savetsky E B Avashia Y J Why social media is transforming plastic surgery Indian J Plast Surg 2020 53 01 4 5 32367910 10.1055/s-0040-1709942 PMC7192656

[10] Wheeler C K Said H Prucz R Rodrich R J Mathes D W Social media in plastic surgery practices: emerging trends in North America Aesthet Surg J 2011 31 04 435 441 21551435 10.1177/1090820X11407483

